# P04.12. Medical practices and attitudes of dual medical license holders in Korea

**DOI:** 10.1186/1472-6882-12-S1-P282

**Published:** 2012-06-12

**Authors:** J Ryu, Y Yun, B Choi

**Affiliations:** 1 Pusan National University School of Korean Medicine, Yangsan, Gyeongnam, Republic of Korea

## Purpose

The collaboration of conventional western medicine (CWM) with traditional Korean medicine (TKM) is a critical issue in the Korean medical system. Becoming dual medical license holders (DMD) having both MD and TKMD licenses is considered a way of overcoming conflicts arising from mutual ignorance and misunderstanding. This study aims to investigate medical practices and attitudes of DMDs who are expected to play an important role in the medical cooperation between CWM and TKM.

## Methods

The questionnaires on the characteristics of the medical practice and attitudes to co-practice were developed and administered to both DMDs and medical students preparing to obtain a second medical license to become DMD. Some items were measured by the five-point Likert scale, ranging from one (strongly disagree) to five (strongly agree). The data of 77 DMDs and 25 students were collected with the help of the Association of DMDs and analyzed.

## Results

Forty-one percent of DMDs have opened medical clinics and Korean medicine clinics simultaneously. DMDs mainly treat musculoskeletal, gastrointestinal, and respiratory diseases in their practices. Co-practice of CWM and TKM is thought to be effective for allergic and endocrine diseases in addition to the three above classifications. They favor CWM modalities in physical examination, laboratory tests, and patients’ education, while TKM modalities are favored in treatment and medication. They believe co-practice is more efficient (3.92) and patients are more satisfied with the co-practice than either CWM or TKM alone (3.88). The inadequate medical insurance system is considered the main obstacle for co-practice (4.34). While contradiction between two medical disciplines is not considered a big problem (2.62), clinical guidelines are highly needed for co-practice (4.15).

## Conclusion

To promote the role of DMDs in developing an integrative medical treatment model, changes in medical legislation and insurance policies seem to be priorities. Research on the cost-effectiveness of co-practice is also required.

